# Physiological Considerations to Support Podium Performance in Para-Athletes

**DOI:** 10.3389/fresc.2021.732342

**Published:** 2021-11-16

**Authors:** Cameron M. Gee, Melissa A. Lacroix, Trent Stellingwerff, Erica H. Gavel, Heather M. Logan-Sprenger, Christopher R. West

**Affiliations:** ^1^Athletics Canada, Ottawa, ON, Canada; ^2^Canadian Sport Institute-Ontario, Toronto, ON, Canada; ^3^Canadian Sport Institute-Pacific, Victoria, BC, Canada; ^4^Faculty of Health Science, Ontario Tech University, Oshawa, ON, Canada; ^5^Faculty of Medicine, International Collaboration on Repair Discoveries, Vancouver, BC, Canada; ^6^Department of Cellular and Physiological Sciences, University of British Columbia, Kelowna, BC, Canada; ^7^Centre for Chronic Disease Prevention and Management, University of British Columbia, Kelowna, BC, Canada

**Keywords:** adapted sports, physical activity, disability, paralympics, exercise

## Abstract

The twenty-first century has seen an increase in para-sport participation and the number of research publications on para-sport and the para-athlete. Unfortunately, the majority of publications are case reports/case series or study single impairment types in isolation. Indeed, an overview of how each International Paralympic Committee classifiable impairment type impact athlete physiology, health, and performance has not been forthcoming in the literature. This can make it challenging for practitioners to appropriately support para-athletes and implement evidence-based research in their daily practice. Moreover, the lack of a cohesive publication that reviews all classifiable impairment types through a physiological lens can make it challenging for researchers new to the field to gain an understanding of unique physiological challenges facing para-athletes and to appreciate the nuances of how various impairment types differentially impact para-athlete physiology. As such, the purpose of this review is to (1) summarize how International Paralympic Committee classifiable impairments alter the normal physiological responses to exercise; (2) provide an overview of “quick win” physiological interventions targeted toward specific para-athlete populations; (3) discuss unique practical considerations for the para-sport practitioner; (4) discuss research gaps and highlight areas for future research and innovation, and (5) provide suggestions for knowledge translation and knowledge sharing strategies to advance the field of para-sport research and its application by para-sport practitioners.

## Introduction

Health and exercise performance have traditionally been considered on a continuum in which the athlete, viewed as the epitome of human physiological performance, is at one end and individuals with disability, traditionally medicalized as a condition to be treated, at the other (1). The para-athlete and the Paralympic Games, encompassing the wider Paralympic Movement, have challenged such dogma. While athletes with disabilities were not unheard of (2), it was the Stoke Mandeville Games (established in 1948) and Paralympic Games (retroactively established in 1960) that have most rapidly advanced para-sport and the Movement (3).

The International Paralympic Committee (IPC; established 1989), recognized as the global governing body of the Movement (4), operates under the vision of enabling para-athletes to achieve sporting excellence. In doing so, the IPC has a classification code that governs the process by which athletes are categorized into a number of groups on the basis of common properties (5). The system aims to determine who is eligible to compete at the Paralympic Games, while ensuring that it is not the degree of impairment but sporting excellence that ultimately determines which athlete or team is victorious (6). Presently, there are 10 eligible impairment types: impaired muscle power, impaired passive range of movement, limb deficiency, ataxia (uncoordinated movement), athetosis (involuntary movements), hypertonia (increased muscle tension), short stature, leg length difference, vision impairment, and intellectual impairment (7).

Though research studies dating back to the 1970s and 1980s have documented exercise responses and the unique physiology of “active” individuals with an impairment, it is only in the last 30 years that research into the “para-athlete” has started to emerge as a field of its own. For example, a search of PubMed databases indicates that in 1990 there were 57 articles on “disability sport” and that by 2020 this number had increased more than twenty-fold. Despite the expansion in research and many excellent para-sport research groups globally there still exists a relative paucity of para-sport research and evidence-based practice—particularly in the physiology domain. There is currently no complete overview of how the different classifiable impairment types impact the physiological response to exercise. Therefore, practitioners within the para-sport field may have trouble incorporating data from isolated case studies and/or studies that focus on a single disability type to develop a comprehensive understanding of how various impairments impact the physiological response to exercise. This lack of cohesive understanding can prevent new practitioners from providing appropriate and timely support of para-athletes and may result in researchers having to spend additional time sourcing background material, rather than designing studies to advance the field.

The present review was written as part of a collaborative effort between Own the Podium and members of the Own the Podium Paralympic Professional Development Working Group that is made up of practitioners, athletes, and academics in Canada. The purpose of this review was to provide a comprehensive overview of the physiological considerations that should be taken into account when supporting para-athletes in applied sports performance roles. Nevertheless, the content of this review is structured in such a way that it will benefit researchers and clinicians who want to gain an overview of para-sport physiology as well those wanting to develop interventions to improve the health and performance of para-athletes. Within this review we discuss the neural control of the physiological response to exercise and the current state of research aimed at enhancing exercise performance in athletes with classifiable impairments under the major sub-groups of limb deficiency, cerebral palsy, SCI, and other classifiable neurological impairments. Whilst athletes with visual and intellectual impairments have practical and biomechanical considerations, they are not expected to exhibit an altered physiological response to exercise or energy requirements and hence are omitted from this review for brevity. Finally, we discuss research gaps and areas of interest for future research and innovation.

## Neuroanatomy of Impairment Groups

**Figure 1:**
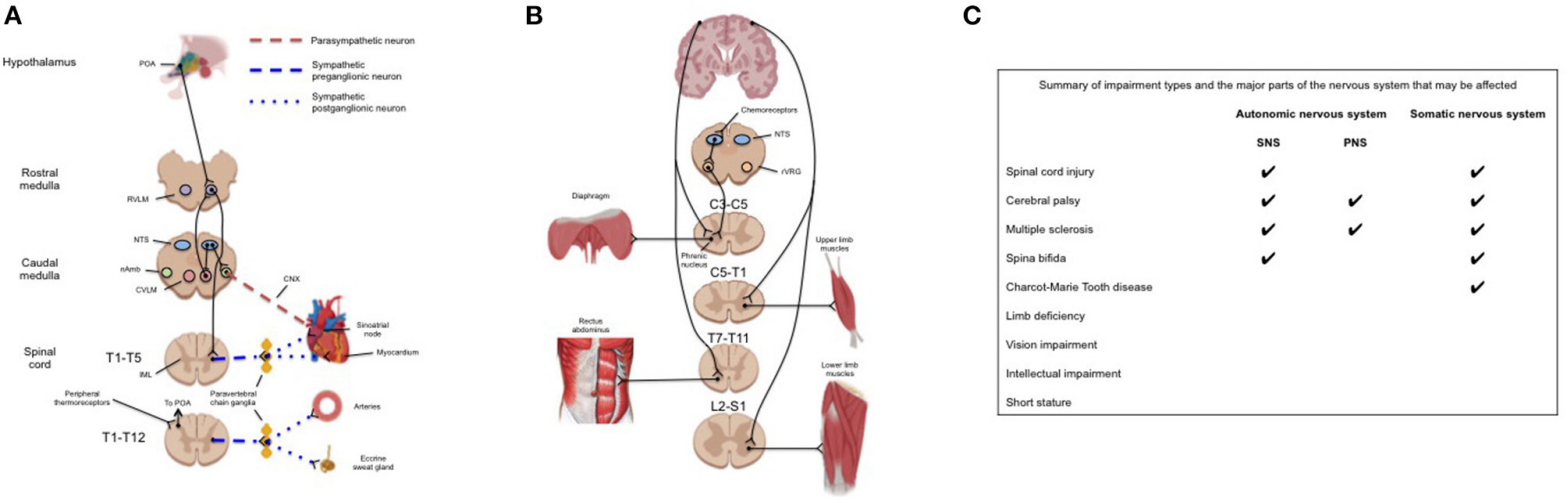
**(A)** Neuroanatomical pathways responsible for the cardiovascular and thermoregulatory response to exercise. Although these responses are integrated within the brainstem the efferent pathways of the sympathetic response pass through the spinal cord and paravertebral chain ganglia. Impairments to these pathways may be at the level of the brainstem (cerebral palsy), spinal cord (spinal cord injury), or peripheral nerves (e.g., multiple sclerosis). **(B)** (Left) Anatomical pathways providing neural drive for the primary muscles of inspiration (diaphragm) and active expiration (rectus abdominus). Pacing of the diaphragm by the rVRG may be overridden by central command and therefore affected by impairments in motor pattern generation (e.g., cerebral palsy); (Right) pathways providing neural drive to upper and lower limb skeletal muscles. **(C)** Summary of common impairment types among para-athletes and which nervous system/s that the impairment may affect. CNX, tenth cranial nerve; CVLM, caudal ventrolateral medulla; IML, intermediolateral cell column; nAmb, nucleus ambiguus; NTS, nucleus tractus solitaries; POA, preoptic area of the anterior hypothalamus; PNS, parasympathetic nervous system; RVLM, rostral ventrolateral medulla; rVRG, rostral ventral respiratory group; SNS, sympathetic nervous system.

Given that many of the abovementioned impairments impact neurological function, it follows that the degree of function is highly variable across impairment groups. This can result in some para-athletes exhibiting an unaffected cardiorespiratory, metabolic, and thermoregulatory response to exercise (e.g., visual impairment), and others exhibiting a severely attenuated cardiorespiratory, metabolic, and thermoregulatory response to exercise compared to able-bodied athletes (e.g., cervical SCI). As such, we believe that para-sport researchers and practitioners should familiarize themselves with fundamental neuroanatomy so as to develop appropriate individualized interventions and understand unique practical considerations of the para-athlete. Figure 1A provides a general guide to neuroanatomy of the autonomic pathways regulating the thermoregulatory and cardiovascular response to exercise. Figure 1B gives an overview of the autonomic and somatic pathways that regulate the ventilatory and skeletal muscle response to exercise. Below we discuss how various impairments may impact the physiological response to exercise in relation to these pathways. It should be noted, however, that there may be a large degree of variability between athletes with the same impairment depending on where the specific lesion (e.g., SCI or cerebral palsy) or injury (e.g., limb deficiency) is located.

## Physiological Response to Exercise Across Impairment Groups

The cardiac (8), vascular (9), ventilatory (10), metabolic (11), and thermoregulatory (12, 13) response to exercise in able-bodied individuals has been reviewed extensively elsewhere and are only referred to here for comparison. Briefly, at the onset of exercise, the sympathetic nervous system (SNS) (Figure 1A) is activated to increase cardiac output and blood pressure to meet the oxygen demands of exercising musculature. Simultaneously, activity of the parasympathetic nervous system (PNS) is gradually withdrawn (14), though present to maximal exercise (14), and increased drive to respiratory muscles (Figure 1B) increases tidal volume to facilitate oxygen supply to, and carbon dioxide removal from, the arterial blood. There also exists a complex interplay between peripheral reflexes (i.e., the baroreflex, chemoreflex and exercise pressor reflex) that provide afferent feedback to the brainstem to “fine-tune” cardiorespiratory, metabolic, and thermoregulatory function (15).

### Spinal Cord Injury

The physiological effects of exercise following SCI have been studied extensively and, for further detail, we refer readers to several excellent reviews (16–18). In short, the physiological response to exercise following SCI is highly variable and dependent upon the severity and level of the injury. Following a “functionally complete” cervical SCI there is altered cardiovascular and sudomotor function, characterized by a reduced peak heart rate (19), an inability to augment stroke volume (20), exercise-induced hypotension (21), and an impaired sweat response that alters evaporative heat loss (22). Whilst the afferent arms for the three major reflexes are intact, the efferent outflow from the brainstem down the spinal cord does not pass through the injury site, and therefore reduces the ability of these reflexes to modulate descending sympathetic circuitry. Interestingly, our group has found that some athletes who have a “functionally complete high-level SCI” can still present with functional sparing in the descending sympathetic fibers (since these fibers are anatomically distinct from the motor/sensory pathways on which the assessment of functional completeness is based) (23). Across sports, we have found that these athletes will consistently out-perform those who do not have functional sparing of these fibers as they can reach a higher exercising heart rate, stroke volume, cardiac output and oxygen uptake (23). In the respiratory system, although neural drive to the diaphragm remains at least partly intact *via* the phrenic nerve, athletes with complete cervical SCI have severely impaired expiratory function due to the loss of neural drive to expiratory muscles (24), which leads to dynamic hyperinflation during higher intensity exercise (25, 26) that can increase dyspnea and have implications for cardiovascular function.

Among athletes who have sustained a complete SCI at the thoracic level, the physiological response to exercise is highly dependent upon the level of the injury. Athletes with complete high-level SCI (i.e., above sixth thoracic spinal level) will have a loss of the ability to vasoconstrict major splanchnic vascular beds, sweat below the level of the lesion, and are likely to have impaired expiratory function (17). However, they may be able to appropriately elevate their heart rate (23) and increase stroke volume (27) *via* direct cardiac sympathetic excitation during exercise. Athletes with complete lower-level thoracic SCI (i.e., below sixth thoracic spinal level) have intact sympathetic drive to the heart and should be able to vasoconstrict and sweat above the level of their injury. The primary muscle of active expiration, the rectus abdominus, should maintain a degree of neural drive and as such these athletes have greater preservation of expiratory function along with practically normal inspiratory function (17).

With respect to alterations at the muscle, it is now relatively well established that SCI causes a fiber type shift in the inactive limbs toward a Type II (i.e., “fast-twitch”) phenotype (28) due to decreases in mitochondrial size and density (29). Despite less favorable conditions for oxygen extraction by the upper limb muscles (30), upper body endurance exercise does appear to result in a number of beneficial adaptations for oxygen off-loading including a muscle fiber type shift toward a greater density of type I fibers (i.e., “slow-twitch”) as well as increased capillarisation and glycolytic enzymes (31). For instance, trained athletes with SCI have significantly higher levels of the oxidative enzymes citric synthase and 3-hyrdroxyacyl-CoA dehydrogenase than untrained and able-bodied individuals, with lower activity of the glycolytic enzyme 6-phophofructokinase reflecting a greater dependence on fat oxidation during exercise (31).

The impact of different levels of complete SCI (i.e., injury to descending sympathetic pathways) on peak physiological responses to exercise is summarized in Table 1.

**Table 1:** Peak physiological responses to arm exercise following complete spinal cord injury relative to able-bodied athletes.

	**Tetraplegia (C5–C8)**	**High paraplegia (T1–T6)**	**Low paraplegia (T7–T12)**
Heart rate	↓	↓ or **=**	↑
	(19)	(23)	(32)
Stroke volume	↓	↓ or **=**	↓
	(20)	(33)	(32)
Cardiac output	↓	↓ or =	?
	(20)	(33)	
Blood press	↓	?	?
	(21)		
Catecholamine release	↓	↓	↑
	(34)	(34)	(34)
Sweat response	↓	↓	?
	(35)	(35)	
Respiratory frequency	**=**	**=**	**=**
	(26)		
Tidal volume	↓	↓	↓
	(26)		
Minute ventilation	↓	↓	↓
	(26)		
Oxygen uptake	↓	↓	↓
	(36)	(36)	(32)

*↓, decreased; =, no change; ↑, increased; ?, unknown*.

### Limb Deficiency

Athletes with limb deficiency, free of other disease or disability, are expected to have an intact central nervous system (CNS) and therefore an unaffected cardiorespiratory and autonomic response to exercise. However, athletes with a limb deficiency (specifically lower limb deficiency performing upper body exercise) can often experience elevated blood lactate concentrations due to a reduced total body mass and high muscle activation in the upper body (37). Due to movement inefficiency, lower limb amputation can increase the energy cost of lower-limb exercise and increase metabolic demand for a given task (38). The limited research available suggests that improving movement efficiency and decreasing the metabolic cost of exercise among lower-limb amputees (38) will likely enhance aerobic exercise capacity.

Athletes with limb deficiency may experience greater thermal strain due to a reduced surface area for evaporative heat loss. Depending on the location of the limb deficiency, and the athletes' use of prostheses, the prostheses may act as an effective insulator and further impair thermoregulation in the remaining limb that is covered by the device. There may also be increased sweat accumulation, and skin breakdown with the use of prosthetics, which may further enhance thermal strain and discomfort. There is evidence that individuals with large surface area of skin grafts may be at higher risk for thermal stress due to the reduction of sweat gland responsiveness and permanent impairment of cutaneous vasodilator capacity of the grafted skin (39).

### Cerebral Palsy

Cerebral palsy is the result of a non-progressive lesion in the developing brain characterized by movement impairments and reduced muscle strength. Among elite level soccer players and cyclists for instance, which are some of the best studied athletes with cerebral palsy, isometric knee extensor strength can be impaired between 31 and 47% (40). Though not in elite athletes, others have shown that voluntary activation can be as low as one-third that of able-bodied individuals (41), suggesting impaired neural recruitment, and that perhaps voluntary strength training may be less effective in this impairment group. In children with cerebral palsy several studies have attributed part of the reduced muscle strength to a type 1 fiber type predominance (41), however we are not aware of such studies in adult or athletic populations. Increased intramuscular fat as well as atrophy and decreased muscle size in the paretic limbs have also been identified as outcomes of cerebral palsy (42).

Athletes with cerebral palsy may present with limited exercise capacity (43) and are at a high risk of musculoskeletal injury, and may additionally experience reduced range of motion, increased muscle stiffness, spasticity, and pain among other medical challenges (44). How the autonomic nervous system functions to meet the physiological response to exercise has not been well-studied (45), however the neural pathways outlined in Figure 1 are expected to remain intact unless there is damage to the hypothalamus—this is also likely true of para-athletes with movement impairments due to acquired brain injuries. Due to impaired co-ordination of movement and the metabolic cost of exercise, metabolic heat production has been found to be higher in children with cerebral palsy compared to able-bodied children for a given workload (46). Others have suggested that this movement inefficiency, coupled with increased muscle tone, may impede the skeletal muscle pump (47) and limit venous return and left-ventricular stroke volume. In children with cerebral palsy, aerobic exercise training may improve movement efficiency and decrease the metabolic cost of exercise—whether this is true for highly-trained athletes, or whether they have reached a “peak” movement efficiency relative to their impairment is unknown. Finally, lung volumes appear to be smaller in adults with cerebral palsy, however it is not known if this is true among athletes (48).

### Other Classifiable Neurological Impairments

Due to the heterogeneity of how other neurological impairments impact the physiological response to exercise, here we only outline the known physiological effects of select impairments not detailed above.

Multiple sclerosis is an autoimmune disease that degrades the myelin sheath of axons within the CNS (49). The most well-known physiological consequence of multiple sclerosis is impaired thermoregulation and occurs when the lesion impacts thermoregulatory centers such as the hypothalamus (50) and is further compounded by reduced sweat gland output in response to thermal stress (51). Other common symptoms of multiple sclerosis relate to muscle weakness, spasticity and fatigue.

Athletes with spina bifida are often included in studies with athletes with SCI, with the primary difference being that it is a congenital birth defect rather than an acquired injury. Spina bifida is most common in the lumbar or sacral spine, in which case sympathetic function is preserved, but may occur in the cervical spine in rare cases (52). Similar to SCI, the effect of spina bifida on physiological responses to exercise is likely a function of the severity of the impairment (i.e., spina bifida occulta, meningocele, or myelomeningocele).

Neurological conditions that effect peripheral nerves (e.g., Charcot-Marie Tooth disease, Guillain Barre syndrome) primarily effect motor and sensory function, including those responsible for pulmonary function, but have the potential to also cause autonomic neuropathy (53).

## Energy Availability and Relative Energy Deficiency in Sport in Para-Athletes

Energy availability (EA) represents the amount of energy left over for optimal physiological function after exercise energy expenditure (EEE) is subtracted from energy intake (EI), and corrected for fat free mass (FFM) (54). It is well established that chronic low EA (LEA) results in myriad negative health, psychological and performance outcomes and is the underpinning etiology of Relative Energy Deficiency in Sport (RED-S) (55). However, laboratory and clinical quantification of EA is impressively difficult, and challenged by methodological considerations that introduce risk for significant under- or overestimation of EI (56) and/or EEE (57); although to our knowledge this has primarily been only examined in able bodied athletes. Indeed, we are not aware of studies that have utilized the “gold-standard” approach of double-labeled water to assess total daily energy expenditure (TDEE). Within TDEE, there is significant work to be done to better understand the EEE demands in para-athletes, of which most data is predominantly in wheelchair athletes. However, even our understanding of EEE in wheelchair athletes is limited. For example, our current compendium of energy costs of physical activities for individuals who use manual wheelchairs is now 10 years old and, despite identifying 266 studies, only 11 studies met the inclusion criteria (58). Only four of these studies included para-athletes, of which 91 were male and 6 female. Given the progress of wheelchair technology advances utilized by modern para-athletes, which would potentially impact gross efficiency outcomes of EEE, much more data needs to be developed to accurately estimate EEE. Therefore, the accurate appreciation of EEE in most classifications of para-athletes remains to be elucidated.

Since the assessment of EA is challenging in evaluating LEA, more chronic indicators of LEA tend to be used for RED-S diagnosis in able-bodied athletes (59), and common outcomes include: clinically low hormones involved in the hypothalamic-pituitary-gonadal-adrenal (HPGA)-axis; amenorrhea; clinically low bone mineral density (BMD); restricted eating leading to disordered eating or eating disorders; increased risk of injuries; poor training adaptations and performance outcomes. However a validated RED-S diagnosis tool in able and para-athletes remains to be developed.

Within para-sport, several recent reviews have highlighted that depending on the impairment many para-athletes have significant challenges with optimizing EI, coupled with potentially altered aspects of EEE, EA, and FFM and thus may be especially at risk for LEA and RED-S (60). Despite over 70 papers published since the 1980's on either RED-S or the Female Athlete Triad in able-bodied athletes (55, 61), we are only aware of three recent publications that have assessed the risk of LEA and/or other chronic indicators of RED-S in para-athletes (62, 63). Depending which assessment tool was used, there were indications of ~73% of elite female athletes with SCI having LEA (62), which was significant more than male athletes with SCI. In another study, 78% of female athletes with SCI were deemed at “risk” for LEA using the LEA in Females Questionnaire (LEAF-Q), although in this study EA assessments were not compromised (63). Taken together and similar to the able-bodied literature, there are considerable discrepancies in RED-S assessment across various tools/parameters, and much more research is required. Among a cohort of male and female track and field para-athletes with cerebral palsy, vision impairment, or limb deficiency >82% had reduced EA and approximately one-third had LEA (64).

The actual prevalence of RED-S may be profound, as a recent survey study in 260 elite para-athletes demonstrated that ~32% had elevated disordered eating questionnaire scores, ~45% of premenopausal females had oligomenorrhea/amenorrhea, ~55% had reported low BMD, but < ~10% had awareness of the RED-S (65). Indeed, athletes with SCI may be especially at risk. For example, wheelchair athletes often have significant lower body muscle atrophy resulting in lower whole-body FFM, which may artificially elevate EA calculations compared to able-bodied athletes. Additionally, ~50% of male athletes with SCI have low testosterone (66), due either to altered HPGA-axis outcomes related to SNS dysfunction and/or aspects of LEA. It is important to note that as of yet, no clinical and laboratory normative LEA data has been published in para-athletes linked to adverse clinical outcomes. Therefore, we have to encourage much more research to develop RED-S specific data and assessment tools in these unique para-athlete populations.

## Physiological Interventions to Optimize Performance

Elite able-bodied athletes and their support staff constantly strive to gain a competitive advantage within the rules of their sport to enhance performance. Two of the more common physiological performance enhancing strategies used by able-bodied athletes may include altitude training (67) and heat acclimation training (68). Although there are extensive research and review articles on these, and other, interventions for the able-bodied athlete, there is often little to no research on how these interventions may affect elite para-athletes—while some may be beneficial, some may be detrimental to performance and/or health.

In recent years there have been a number of studies that highlight the impacts of respiratory muscle training (69, 70), abdominal binding (71), and heat acclimation (72, 73) in para-athletes (see 23). However, there remains a large gap in the research and applied knowledge for how these protocols may translate to benefit para-athletes in all impairment groups. It is beyond the scope of this review to fully review these interventions. Nevertheless, some key interventions and practical considerations to working with para-athletes are highlighted in Tables 2 and 3, respectively.

**Table 2:** Key physiological interventions specific to para-athletes.

**Key interventions**	**Existing evidence by athlete group**	**Considerations for other para-athlete groups that may benefit**
Respiratory muscle training	In able-bodied endurance athletes, IMT appears to benefit performance by delaying diaphragm fatigue, offsetting the respiratory muscle metaboreflex, and attenuating respiratory discomfort (10, 74)	• Among endurance athletes with minimal impairment to cardiorespiratory function (i.e., limb deficiency, intellectual and visual impairment), competing in para-sports with high ventilatory demand, there is likely potential benefit from similar IMT protocols as those applied to able-bodied athletes
	In athletes with cervical SCI, pressure threshold IMT (69), and combined IMT and EMT (70) (30 repetitions performed twice daily on 5 days/week) appear to ↑ respiratory muscle strength and aerobic exercise capacity	• No respiratory muscle training program has been assessed in athletes with cerebral palsy. However, it is plausible that strengthening the respiratory muscles could elicit functional and structural adaptations that benefit trunk stability and movement patterns—as has been observed in children with cerebral palsy (75)
		• Combined IMT and EMT has not been examined in athletes with multiple sclerosis or muscular dystrophy, however evidence from the non-athletic population suggests it can reduce self-reported fatigue and the severity of breathlessness, respectively (76, 77)
Abdominal binding	In athletes with cervical SCI, can prevent exercise induced hypotension, prevent pooling of blood in the abdomen and acutely ↑ resting cardiac output (78), ↓ exercising lung volumes (25), and enhance both lab- (25) and field-based (71) exercise performance	• The efficacy of abdominal binding among athletes with other impairments has yet to be assessed however, theoretically, it may improve central hemodynamics in athletes with paraplegia who have impaired neural drive to the rectus abdominus (see Figure 1B) and may enhance stability among athletes with impaired muscle power or co-ordination of the trunk musculature
Heat acclimation	In able-bodied athletes, or para-athletes with an intact SNS: ↓heart rate, ↑cutaneous blood flow and sweat rates, ↓core temperature and improved exercise performance Secondary benefits, may include ↑ plasma volume and ↑stroke volume (68)	• There is potential for athletes with minimal impairment to autonomic pathways, vasomotor and sweat control to benefit from similar physiological responses to heat acclimation as shown in able-bodied literature. Such athletes would likely benefit most from protocols similar to those that have been most effective in able-bodied athletes (79)
		• Some athletes with a SCI and MS have significant challenges in the heat due to poor sweat rates and poor thermoregulation and therefore need enhanced monitoring around optimizing hydration practices. Athletes with MS are especially more sensitive to heat, and need heat mitigating strategies. As such, we suggest that these athletes are closely supervised by a practitioner if undergoing heat acclimation training
	In athletes with high risk of thermal strain and/or impaired SNS, potential improvements in heat tolerance, pacing strategies, and ↑ in plasma volume	• Heat acclimation/acclimatization may be recommended to improve performance and heat tolerance for athletes competing in hot-humid environments, sports requiring a high aerobic demand and athletes with a high risk of thermal strain
	In athletes with MS, the physiological response and performance benefits of heat acclimation may not outweigh the negative impacts it has on symptoms (50)	• Symptoms including early onset of fatigue associated with MS may be exacerbated by as little as a 0.5°C increase in core temperature in 60–80% of MS patients. In athletes with MS it is important to limit their exposure to the heat, however, more research is needed to better understand if their symptoms and heat tolerance may improve with heat acclimation training
Altitude Training	Living and/or training at altitude can enhance aerobic exercise capacity, primarily *via* augmentation of red blood cell count, in elite and sub-elite able-bodied athletes (67)	• Among endurance athletes with minimal impairment to cardiorespiratory function (i.e., limb deficiency, intellectual and visual impairment), competing in para-sports with high aerobic demand (e.g., track event of 5,000 m and greater or their equivalent), there is likely potential benefit from similar altitude training protocols as those proven effective for able-bodied athletes. However, this remains to be established in para-athletes
		• Given the risks associated with low oxygen availability at altitude and the limited research in para-athletes with oxygen transport limitations we suggest para-athletes are closely supervised by a practitioner if undergoing altitude training
	In able-bodied athletes, the use of sildenafil, has been shown to ↑ exercising peak power output and peak oxygen uptake at high altitudes above 4,000 m	• The use of sildenafil would not be recommended prior to competition and athletes who are prescribed sildenafil (commonly used to treat erectile dysfunction in athletes with SCI) should be aware of the potential negative effects on exercise performance
	In athletes with SCI, best evidence suggests that sildenafil does not enhance exercise capacity compared to placebo at sea level or altitude (80)	• No major global able-bodied or para-athletes specific competitions are held at altitudes above 4,000 m

*CP, cerebral palsy; EMT, expiratory muscle training; IMT, inspiratory muscle training; MS, multiple sclerosis; SCI, spinal cord injury; SNS, sympathetic nervous system*.

**Table 3:** Practical and special considerations for supporting para-athletes.

**Special training considerations**	**Specific sub-factors related to para-athletes**	**Physiological and applied practical considerations**
Environmental conditions	Medications, sleep deprivation and fitness levels may impact an athlete's heat tolerance	• Medication use and sleeping habits should be monitored when the athlete is traveling or competing in a warm environment. Ensure athletes aerobically fit to tolerate training/competing in the heat
	Equipment interactions and sweat hygiene	• Maintain consistent prosthetic hygiene to decrease risk of infections, skin breakdown due to accumulation of sweat or contact with sports equipment especially during hot humid conditions where sweat rates may be higher
		• Trial equipment and prosthetic fit prior to competing in altered environmental conditions
	Early onset of fatigue ↓ in performance in hot or cold environments	• Use of cooling strategies in warm environments (slushies, ice vests, cold water immersion, cold spray, menthol) is recommended for all athletes with impairments (81)
		• Heat acclimation training to improve heat tolerance (refer to Table 2)
		• Warming strategies in cold environments (warm fluids, extra layers, heated garments). Consider the effects of decrease blood flow, and sensation
	Pace awareness and perception of effort are exacerbated in the heat (43, 46)	• Athletes with CP, intellectual impairment or VI who compete without a guide, should practice pacing outcomes in the target weather conditions prior to competition to establish a pre-determined pacing strategy based on the ambient conditions
	↑ Risk of thermal strain; ↓ evaporative or convective heat loss, ↑ metabolic heat, ↓ vasomotor and sweat control	• Athletes with a SCI, limb deficiency, CP, VI, short stature, or other neurological conditions would benefit from individualized internal and external cooling strategies (pre, per and/or post cooling based on the sport)
		• Consider the cost benefit of the added thermal strain when using additional clothing garments, equipment and wearable devices
	Cold environments may impact athletes with muscle stiffness, nerve pain, changes in vasomotor and sudomotor tone	• Ensure the temperature in the gym and training facilities are a neutral temperature. If you are training or competing in cold environments, ensure the athlete has a good warm up and potentially look to pre warming techniques to minimize the cold related symptoms
Monitoring	Pace awareness and perception of effort (43, 46)	• In athletes with an intellectual impairment, using RPE scales may not be appropriate
		• For athletes with intellectual or visual impairment, consider strategic use of a pacer in practice, followed by trialing without the use of a pacer in practice, to mimic race/competition demands
	RPE for monitoring	• May consider using a differentiated approach for RPE relative to central (cardiorespiratory), peripheral (blood lactate), and overall (central + peripheral) feeling of effort (82)
	Impaired peak heart rates	• In athletes with impaired SNS function, monitor training using heart rates based on the individual athletes peak exercising heart rates
	Impaired skin conductivity	• In athletes with skin grafts or neurological conditions it may not be appropriate to use finger or wrist worn heart rate monitors due to decrease skin conductivity
	↓ Blood lactate clearance	• Athletes with a lower limb deficiency performing upper body exercise may have reduce blood lactate clearance due to a decrease in total body mass and increased activation of upper body musculature
Training considerations	Consider ADL's (transferring, driving, pushing, cooking, bowel routines etc.)	• ADL's should be a consideration in programming overall training workloads, as these may influence fatigue and readiness for training to a greater extent than able-bodied athletes
		• In athletes with SCI, lower limb deficiency, and VI the workload completed in training can have a big impact on what the athletes can do for the remainder of their day as well.
	↑ in spasticity following maximal exertion due to an overexcitability of the stretch reflex Sensitivity to the stresses incurred by training sessions with high anaerobic content	• For athletes with hypertonia, ensure appropriate recovery times and balanced training with high intensity/ high anaerobic efforts when planning training phases. May be at risk of increased hypertonia, pain, stiffness, and clonus
Travel	Sleep disorders, altered distribution of melatonin and temperature regulation throughout the day	• Jet lag and travel fatigue may be exacerbated in athletes with sleep disorders and athletes with VI due to an already disrupted circadian rhythm. Establish a travel plan, a sleep schedule and periodized training upon arrival pre-event following long haul trips
		• In athletes with intellectual and visual impairments, and some athletes with a SCI, sleep medications and poor sleep impact on circadian rhythms and optimal hormone regulation (e.g., lowered testosterone, increased cortisol), which can impact on eating behaviors and body composition outcomes
	Athletes may dehydrate themselves during travel and may go multiple days without emptying their bowels	• Establish an individualized hydration plan during travel and upon arrival. Consider the athletes bowel routines when planning training schedule upon arrival • Wheelchair users are at increased risk for dehydration, especially when traveling, due to accessibility challenges
	↑ stiffness or spasticity with long international travel	• Symptoms may be exacerbated with long haul travel for athletes with increased spasticity, decreased range of motion and movement
		• Promote movement during travel as much as possible, bring their own seat cushions for the plane and encourage weight shift. Focus on mobility and light movement in the first few days of arrival, allow time for the athletes to lay and stretch out upon arrival
	Stump volumes change due to the accumulation of fluid if the prosthetic limb is removed in flight	• Awareness and regular stump care. Proper fitting of prosthetics and having alternative training strategies when tissue health is compromised

*ADL, activity of daily living; CP, cerebral palsy; RPE, rate of perceived exertion; SCI, spinal cord injury; VI, visually impaired*.

## Future Areas of Research and Innovation

The Tokyo 2020 Summer Paralympic Games featured 4,403 athletes from 162 participating countries who competed in a total of 539 events were contested across 22 sports (83). Despite growing interest and participation in Paralympic sport, along with the influx of published articles on disability sport over the last quarter century, there remains a lack of evidence-based physiological interventions to improve performance in the para-athlete, especially those without SCI. This is in part due to the barriers associated with conducting research on small heterogenous cohorts of para-athletes. For example, Stephenson et al. (73) conducted a heat acclimatization intervention in seven elite para-triathletes across five different impairment groups. While conducting such a research study is commendable from a logistical perspective, the heterogeneity of the cohort makes it difficult to form conclusions as to how the intervention may be applied to para-athletes with various impairments. To overcome these limitations, practitioners often rely on knowledge transfer from experience, colleagues, and anecdotal evidence through case study approaches to inform their practice. Current practice is often adapted based on able-bodied research, yet there is little validated data on how many of the interventions, protocols, training methods and assessment tools used by practitioners directly translate to support athletes in all impairment groups or across athletes with varying severities of impairment (i.e., SCI level, hemiplegia vs. diplegia, level of amputation). Furthermore, there is a distinct lack of published data on the physiological demands of para-sports from which practitioners can base physiological interventions and training programs. This includes simple, relatively non-invasive data such as game duration/intensity profiles integrated with heart rate, oxygen consumption, and/or lactate profiles.

Thermoregulation is an area that has been relatively well researched in para-athletes. However, the majority of research focuses on athletes with a SCI due to the known higher risk of thermal strain in this population (22). Therefore, we can only hypothesize as to whether other impairment groups would experience increased thermal strain relative to able-bodied athletes, or would benefit from cooling strategies and/or heat acclimation training. The current literature on heat acclimation is limited in elite para-athletes (72, 73). This not only highlights the difficulty in recruiting large homogeneous samples of para-athletes but emphasizes the need to determine impairment specific physiological responses, adaptations, and performance outcomes to training in the heat for practitioners to individualize training preparation for Paralympic athletes.

Competing in the heat is not the only environmental condition that impacts para-athletes. We believe that further research should also examine the impact air quality, cold environments, and altitude on performance. In particular, altitude training is a common intervention adopted by elite able-bodied endurance athletes (84), but we are unaware of any altitude training interventions in para-athletes whom commonly compete at moderate altitudes. Additionally, the areas of travel and immune function are only starting to emerge in conversation and the scientific literature (85) and warrant further research.

It is understood that elite para-athletes have a high prevalence of injury and illness (86), and is therefore imperative for the integrated support staff to first support the para-athletes health and well-being for them to train and perform at the highest level. There is a plethora of data indicating the negative health, psychological and performance impacts that LEA has on able-bodied athletes (87), however, future research is critical to understanding the prevalence and consequences of LEA and metabolic considerations across all impairment groups. Currently, a challenge for practitioners is the accuracy and validity of assessment tools available for monitoring EA, as all practical methods were developed with no consideration of athletes with an impairment. Even though many para-athletes may be at high risk for developing RED-S, we have no normative data or valid assessment tools to accurately monitor resting metabolic rate, EE, and RED-S in athletes with an impairment.

## Conclusions

The present review has outlined the neurophysiology of the most common impairment groups and the practical considerations when supporting para-athletes along with performance enhancing interventions. We acknowledge that a highly individualized approach to supporting para-athletes is needed due to the variability not only between but within impairment groups and emphasize the need to further enhance our approach to providing practitioners with evidence-based research. We suggest that this may be achieved by various knowledge translation strategies, including (i) modules to educate current and future practitioners in the para-sport field, (ii) encouraging para-sport practitioners to publish and/or present on unique field observations, and/or (iii) the sharing of data between sport systems including cross collaborative projects between research groups from different nations as well as sports with para-athletes of similar impairment groups.

## Author Contributions

All authors provided substantial contributions to the conception and design of the work and drafting the work or revising it critically. Final approval of the version submitted/published and consent for publication has been agreed by all authors.

## Funding

CG and EG were supported in the writing of this manuscript by funding from Own the Podium.

## Conflict of Interest

The authors declare that the research was conducted in the absence of any commercial or financial relationships that could be construed as a potential conflict of interest. The reviewer EB declared a past co-authorship with one of the authors TS to the handling editor.

## Publisher's Note

All claims expressed in this article are solely those of the authors and do not necessarily represent those of their affiliated organizations, or those of the publisher, the editors and the reviewers. Any product that may be evaluated in this article, or claim that may be made by its manufacturer, is not guaranteed or endorsed by the publisher.
